# Deviations in psycho-sexual development of teenage girls and its legal consequences in modern society

**DOI:** 10.1192/j.eurpsy.2021.2157

**Published:** 2021-08-13

**Authors:** L. Baranskaya

**Affiliations:** Psychiatry, Psychotherapy And Narcology, Ural State Medical University, Yekaterinburg, Russian Federation

## Abstract

**Introduction:**

When studying the multiple aspects of the problem of criminal offences of a sexual character committed by adult males towards teenage girls, the role of digital communication technologies is not taking into account.

**Objectives:**

To reveal the negative aspects of social and family connections and the influence of digital communications on the formation of deviations in psycho-sexual development of teenage girls who had become victims of sexual delicts of a non-violent character.

**Methods:**

We have studied the specifics of psycho-sexual behavior of seventeen teenage girls, aged 12-16 and their Internet and SMS correspondence with adult males by analysis of semantics and pathos-psychological markers.

**Results:**

Intellectually, all the teenage girls are within normal age limits. They were bringing up in full, materially well-off families. Most of the girls’ parents experience a formal attitude towards them. The disrupted emotional ties of the teenage girls with mother or both parents leads to deviations in the development of normal teenage reactions, sexual attitudes. Their feeling of loneliness within the family, forces them to turn towards support to Internet-society or other adults of the opposite sex to their parents’ acquaintances. The desire to ascertain perfectionist expectations and self-assertion leads the teenagers to the realization of various forms of auto-destructive sexual behavior. They actively demonstrate in the Internet obscene photos of their genitals. While trying out their sexual importance, they persistently urge adult males towards sexual contacts.

**Conclusions:**

Thus, the negative aspects of psycho-sexual development of teenage girls can disrupt their sexual behavior in adulthood.

**Disclosure:**

No significant relationships.

